# High‐fat diet aggravates the severity of the in vitro posttraumatic osteoarthritis model through macrophagic FBW7

**DOI:** 10.1002/iid3.988

**Published:** 2023-09-26

**Authors:** Lijun Duan, Yuan Ma, Chen‐Guang Feng, Xing Yu

**Affiliations:** ^1^ Department of Orthopedics SijiQing Hospital Beijing People's Republic of China; ^2^ Department of Orthopedics Bayannaoer City Hospital Bayannaoer City Inner Mongolia People's Republic of China; ^3^ Department of Clinical Medicine Inner Mongolia University of Science and Technology Inner Mongolia People's Republic of China; ^4^ Department of Orthopedics, Dongzhimen Hospital Beijing University of Chinese Medicine Beijing People's Republic of China

**Keywords:** FBW7, high‐fat diet, macrophages, osteoarthritis

## Abstract

Osteoarthritis (OA) is a prevalent and distressing chronic degenerative joint disorder characterized by damaged articular cartilage and inflamed joints. Among risk factors, obesity has emerged as the second‐leading contributor to OA after age. Obesity is believed to play a key role in the development and progression of OA. This study aimed to investigate the role and underlying mechanisms of high‐fat diet (HFD)‐induced obesity in the development of OA. Our findings revealed that HFD could aggravate the destabilization of the medial meniscus (DMM)‐induced damage in the mouse model of obesity. Similar results were observed when macrophages obtained from HFD‐fed mice were cocultured with cartilage and subsequently stimulated with interleukin‐1β (IL‐1β). Mechanistically, we observed a decrease in the expression of intraarticular macrophagic FBW7, which was implicated in the aggravation of OA in the HFD‐fed animal. Furthermore, by modulating the immune status of macrophages, we found that reversing the macrophagic expression of FBW7 in these cells can alleviate the chondrocyte damage. In conclusion, this study provides novel insights into the pathological mechanisms underlying HFD‐related OA development by identifying the role of FBW7 in synovial macrophages. These findings open up new avenues for research and therapeutic interventions targeting HFD‐related OA.

## Graphical abstract

High‐fat diet (HFD) aggravate the osteoarthritis (OA) damage caused by destabilization of the medial meniscus (DMM) in mice. Decreased intraarticular macrophagic FBW7 expression involved in the aggravate OA severity in HFD animal. This study provides a novel insight into the pathological mechanisms of HFD‐related OA pathegenesis.

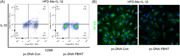

## 1 INTRODUCTION

Knee osteoarthritis (OA) is the most prevalent degenerative joint disease, characterized by symptoms including pain, stiffness, swelling, and limited knee joint movement, significantly affecting lower limb mobility.^1^, ^2^ Recent epidemiological data show that the global population of patients with OA has exceeded 0.3 billion, leading to significant consumption of healthcare resources, reduced work capacity, diminished quality of life, and imposing a significant direct and indirect economic burden.^3^, ^4^, ^5^, ^6^ The aging population and lifestyle changes have contributed to a continual rise in knee OA cases annually, exacerbating the disease and imposing increasingly severe physical, economic, and societal consequences.

Apart from aging, obesity is a well‐recognized and prevalent risk factor for knee OA.^7^ The modern lifestyle often includes a high‐fat diet (HFD), which disrupts metabolic processes and leads to an abnormal increase in blood lipids or lipoproteins. The impact of a continuous HFD on knee OA has gained increasing attention in this field. Inflammatory responses are considered significant pathogenic mechanisms of knee OA.^8^ The disruption of lipid metabolism induced by HFD results in the production of excessive oxidized low‐density lipoprotein and free fatty acids, which in turn stimulate the expression of inflammatory factors in the joint cavity.^9^ Prolonged consumption of HFD disrupts the lipid metabolism balance in articular cartilage, leading to metabolic changes and chondrocyte dysfunction, ultimately resulting in cartilage damage.^10^, ^11^ Furthermore, HFD has been found to contribute to the aggravation of joint injuries in arthritis.^12^ Although it is generally understood that lipid metabolism affects the metabolic homeostasis of articular cartilage, few studies have explored the specific mechanisms by which HFD aggravates knee OA.

The aggravation of arthritis injuries due to HFD suggests the involvement of immune‐related mechanisms. Macrophages, as key immune cells in the joint cavity, are sensitive to changes in lipid metabolism.^13^, ^14^ In this study, we stimulated the hyperlipidemia state by subjecting mice to an 8‐week HFD. During this period, direct damage to the knee OA from the HFD itself did not occur. Through the in vivo and in vitro OA modeling experiments, we sought to reveal how HFD contributes to the aggravation of arthritis. The goal is to provide new insights and a theoretical foundation for understanding the etiology of HFD‐related OA and developing effective intervention strategies.

## 2 MATERIALS AND METHODS

### 2.1 Animal models

In this study, we employed the destabilization of the medial meniscus (DMM) surgical procedure to establish the OA model, as described in previous studies.^15^, ^16^, ^17^ The right knee of mice underwent DMM surgery to induce OA, while the left knees were sham‐operated. A xylazine/ketamine solution was injected intraperitoneally to anesthetize the animals. After anesthesia, the medial skin of the hindlimbs was disinfected using iodophor and alcohol. An aseptic medial capsular incision was made to expose the knee joint, and then the medial menisco‐tibial ligament was resected to dislocate it. Before the DMM surgery, mice followed either a routine diet or HFD for 8 weeks.^18^ The Sham and DMM alone groups were provided with the routine diet, whereas the Sham + HFD and DMM + HFD groups were given the HFD. After 4 weeks of DMM surgery or being on HFD, the mice were euthanized. Synovial fluid from the joints was collected by thorough washing with PBS, and macrophages were isolated. The Osteoarthritis Research Society International (OARSI) scoring system was employed to evaluate the severity of OA in the medial femoral condyle and medial tibial plateau, as previously described.^16^, ^17^ Briefly, the OARSI scoring system involves the analysis of articular cartilage in two representative sections from each stained slide to assess the pathological condition of each joint. Scores ranged from zero (indicating unchanged articular cartilage) to six (indicating severe OA). The OARSI score was evaluated in a blinded fashion by two independent graders. Ethical considerations were strictly followed in our experiment, which was supervised and approved by the Animal Ethics Committee of our hospital.

### 2.2 Cell culture and transfection

Primary chondrocytes were isolated from the knee cartilage of mice as previously described.^19^ Mouse macrophage cell line RAW 264.7 and chondrocytes were cultured in RPMI‐1640 medium supplemented with 10% fetal bovine serum (FBS) at 37°C and 5% CO_2_. Lentivirus vectors carrying FBW7 overexpression constructs, as well as negative control vectors (ITS), were obtained from GenePharma. For the coculture experiments, macrophages were cultured in the upper compartment, while chondrocytes were cultured in the lower compartment.

### 2.3 CCK‐8 assay

After transfection, cells were seeded into a 96‐well plate at a density of 1 × 10^3^ per well. Experiments were conducted in triplicate. The plate was placed in the incubator for 1 day. Following that, 10 μL of CCK‐8 solution was added to each well, and the plate was incubated for an additional 2 h. Subsequently, the optical density (OD) value at 450 nm was measured using a microplate reader to calculate the cell proliferation inhibition rate.

### 2.4 Determination of the levels of inflammatory factors

The levels of inflammatory factors in the cells were determined using the corresponding kits provided by Nanjing Jiancheng Bioengineering Institute (Nanjing, China).

### 2.5 Hematoxylin and eosin (H&E) staining

Knee joints were subjected to H&E staining to visualize their general morphology. The washed knee joint tissues were fixed in a 4% neutral formaldehyde solution for 24 h to denature. Following fixation, the tissues were dehydrated with gradient alcohol and placed in the embedding machine. The molten wax was poured into the embedding machine, where tissues were labeled before the wax solidified. The wax block containing the tissues was frozen at −20°C, trimmed and sectioned into 4 μm slices, and baked at 60°C. The baked tissue sections were subjected to a series of steps, including immersion in xylene, followed by rehydration through ethanol. The sections were then stained with hematoxylin, differentiated with 1% hydrochloric acid alcohol, and subsequently immersed in alcohol and stained with eosin. Finally, the tissues were immersed in 95% alcohol, a gradient of ethanol solutions, soaked with xylene, and sealed with neutral gum.

### 2.6 Quantitative reverse transcription polymerase chain reaction (qRT‐PCR)

TRIzol reagent (Beyotime) was used to extract and purify RNA from tissues and cells. The extracted RNAs were then reverse‐transcribed into complementary DNA (cDNA) using the PrimeScript RT kit (Takara). Subsequently, cDNA was amplified using the SYBR Premix Ex Taq II kit (Takara). The amplification conditions were as follows: 95°C for 10 min (45 cycles), 95°C for 15 s, 60°C for 20 s, and 72°C for 20 s. The gene expression levels were calculated using the $2^{‐\Delta \Delta C_{T}}$ method, with GAPDH serving as the internal reference gene for normalization.

### 2.7 Flow cytometry

The cells (1 × 10^6^ cells) were treated with V‐FITC and PI from the FITC Annexin V Apoptosis Detection Kit (Beyotime) at 4°C for 15 min. Then, the cells were washed three times with prechilled phosphate‐buffered saline (PBS) before being suspended in the buffer for further analysis. A total of 40 μL of each diluted fluorescent antibody working solution (anti‐CD68‐PE and anti‐IL‐33‐FITC, both at a dilution of 1:200, BioLegend, Inc.) was added to each cell sample. The cells were incubated at 4°C for 30 min in the dark. After incubation, 200 μL of FACS buffer was added to each sample. The mixture was centrifuged at 1000 rpm for 10 min at 10°C, and the supernatant was discarded. The cell samples were analyzed using a FACSCalibur flow cytometer and CellQuest software (Becton Dickinson).

### 2.8 Western blot

The total protein was extracted using radio‐immunoprecipitation assay lysis buffer (Beyotime). Equal amounts of protein were dissolved in sodium dodecyl sulfate‐polyacrylamide gel electrophoresis (SDS‐PAGE) loading buffer, separated by electrophoresis, and transferred onto polyvinylidene fluoride (PVDF) membranes (Pall Life Sciences). The PVDF membranes were then blocked with 5% skim milk at room temperature for 1.5 h. After blocking, the membranes were washed and incubated overnight at 4°C with the corresponding specific primary antibodies. Subsequently, the membranes were washed and incubated with secondary antibodies at room temperature for 1 h. Eventually, the immunoblot signals were detected using the Plus chemiluminescence detection kit (Amersham Biosciences). The relative gray density of protein bands was determined using ImageJ software (ImageJ 1.5, NIH).

### 2.9 Immunofluorescence

For immunofluorescence assays, the knee joint tissues were fixed with 4% paraformaldehyde. After fixation, the tissues were permeabilized using a PBS solution containing 0.5% Triton X‐100 (Beyotime Biotechnology). To block nonspecific binding, QuickBlock^TM^ blocking buffer from the Immunol Staining Kit (Beyotime Biotechnology) was applied. The tissue sections were then incubated with the selected primary antibodies, followed by incubation with the corresponding fluorescent secondary antibodies. Subsequently, the sections were washed thrice with PBS, and the nuclei were stained with (4′,6‐diamidino‐2‐phenylindole [DAPI]; Yeasen Biotechnology Co., Ltd.). The sections were visualized, and images were captured using a fluorescence microscope (Olympus Corporation).

### 2.10 TUNEL staining

The One‐Step terminal deoxynucleotidyl transferase‐mediated dUTP nick‐end labeling (TUNEL) Kit (Beyotime) was used to assess cell apoptosis. Briefly, lung adenocarcinoma cells were rinsed with PBS and fixed with 4% paraformaldehyde. Subsequently, cell nuclei were stained with DAPI. The apoptotic cells were detected using a fluorescence microscope (Olympus).

### 2.11 Statistical analysis

Statistical analyses were performed using GraphPad Prism 6.0 (GraphPad Software). Data were expressed as mean ± SEM. For comparisons between the two groups, the Student's *t* test was used. For comparisons among multiple groups, one‐way analysis of variance (ANOVA) was employed, followed by the Tukey post hoc test for multiple comparisons. *p* values less than 0.05 were considered statistically significant.

## 3 RESULTS

### 3.1 HFD aggravates posttraumatic OA outcomes in mice

**Figure 1:**
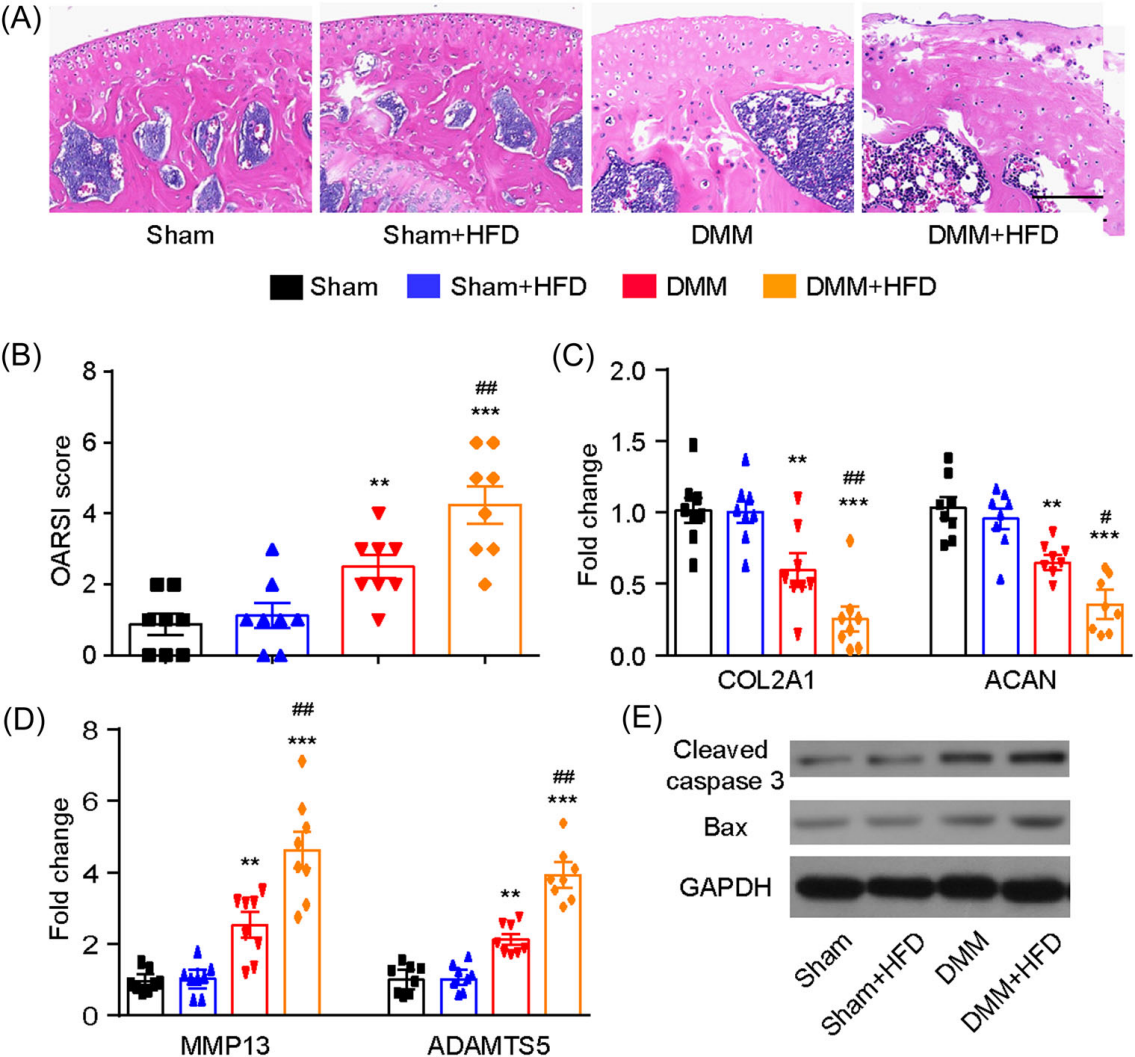
High‐fat diet (HFD) mice present aggravated pathological changes in joints. (A) Representative images of hematoxylin and eosin (HE)‐stained sections of knee joints from each group. *N* = 6 per group. Scale bar: 100 µm. (B) The severity of articular cartilage damage was evaluated using the Osteoarthritis Research Society International (OARSI) scoring system. (C, D) Quantitative polymerase chain reaction (PCR) analysis of COL2A1, ACAN, MMP13, and ADAMTS5 in each group. (E) Protein expression levels of cleaved caspase and Bax in chondrocytes were measured by western blot analysis. *N* = 8, one‐way analysis of variance (ANOVA) followed by Tukey post hoc test, *p < .01, ***p < .001 compared with Sham; #*p* < .05, ##*p* < .01 compared with destabilization of the medial meniscus (DMM).

We randomly assigned male mice into four groups to investigate the effects of HFD on arthritis development and outcome in an experimental DMM‐induced OA model. Histologically, the articular cartilage of mice in each experimental group was stained with H&E. The cartilage tissues of the Sham + HFD group exhibited a nearly normal morphological structure, while the cartilage tissues of the DMM + HFD group displayed more severe destruction compared with the DMM group 4 weeks postsurgery (Figure 1A). The histological findings were consistent with the OARSI score, which revealed that HFD‐treated injured joints had significantly higher cartilage scores compared with vehicle‐treated injured joints (Figure 1B). We further investigated the effects of HFD on chondrocyte homeostasis. The articular cartilage of HFD‐fed mice showed consistently decreased levels of COL2A1 and ACAN and increased levels of MMP13 and ADAMTS5 (Figure 1C,D). These results were confirmed by western blot analysis, which indicated increased expression of the proapoptotic proteins cleaved caspase‐3 and Bax in response to HFD (Figure 1E).

### 3.2 The impact of HFD on the chondrocytes was evaluated in coculture with macrophages in vitro

**Figure 2:**
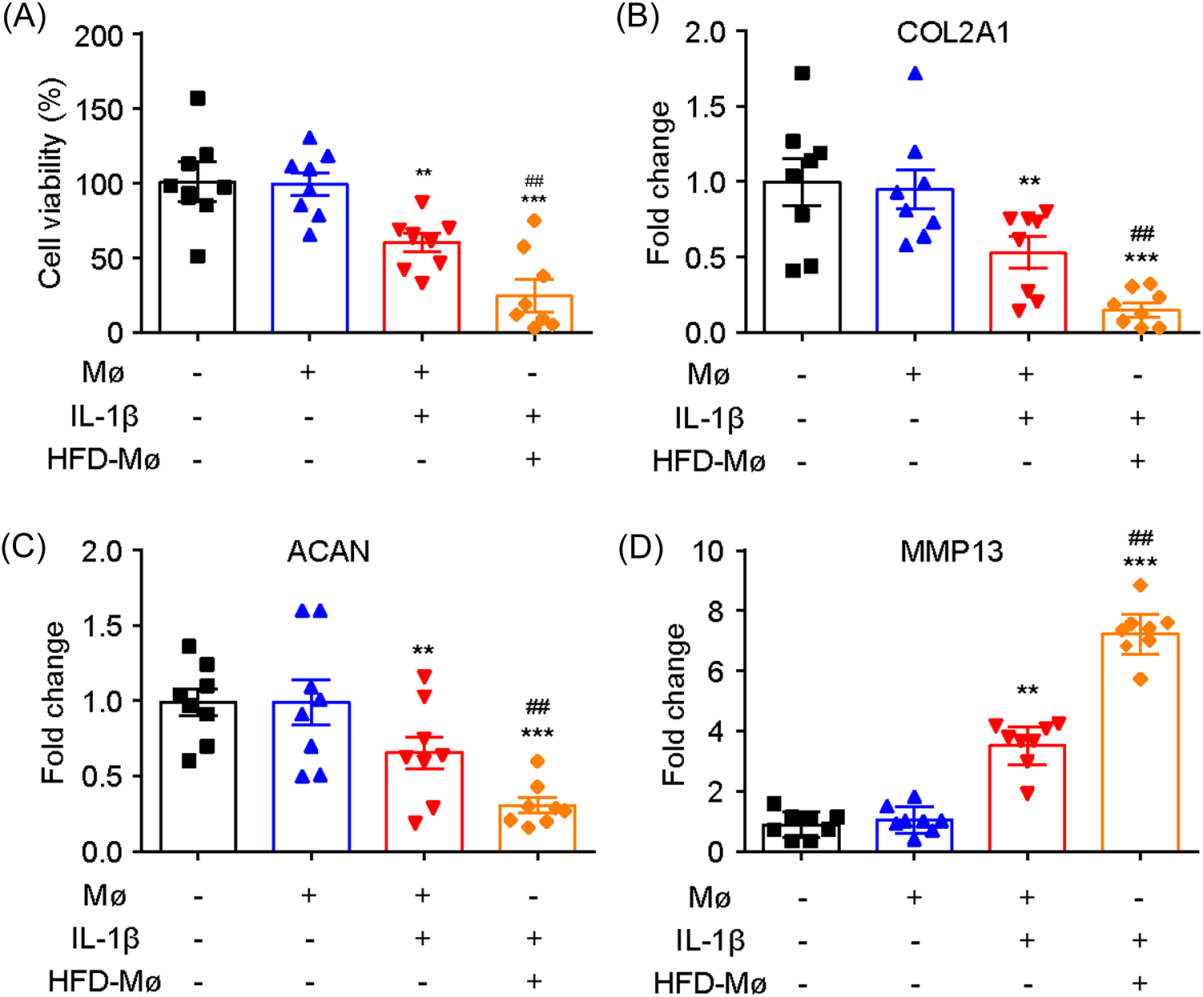
A transwell coculture system was used to determine the effects of high‐fat diet (HFD) exposure on chondrocyte injury. (A) The effect of macrophages isolated from HFD mice on interleukin‐1β (IL‐1β)‐induced chondrocyte injury as examined by cell viability. (B–D) The effect of macrophages isolated from HFD mice on IL‐1β‐induced chondrocyte injury as examined by quantitative polymerase chain reaction (PCR) analysis of COL2A1, ACAN, and MMP13. *N* = 8, one‐way analysis of variance (ANOVA) followed by Tukey post hoc test, **p < .01, ***p < .001 compared with Mø; ##*p* < .01 compared with Mø + IL‐1β.

To further confirm these findings, additional in vitro experiments were conducted. Macrophages were extracted from control or HFD‐fed mice, and they were cocultured with chondrocytes. Initially, the addition of macrophages to the coculture had no significant effect on chondrocyte viability. However, when stimulated with interleukin‐1β (IL‐1β), the viability of chondrocytes decreased. Notably, coculturing chondrocytes with macrophages extracted from HFD‐fed mice further exacerbated the cell injury (Figure 2A). The results obtained from qRT‐PCR analysis of COL2A1, ACAN, and MMP13 gene expression aligned with the cell viability data (Figure 2B–D).

### 3.3 Macrophagic FBW7 levels were decreased in HFD‐aggravated posttraumatic OA

**Figure 3:**
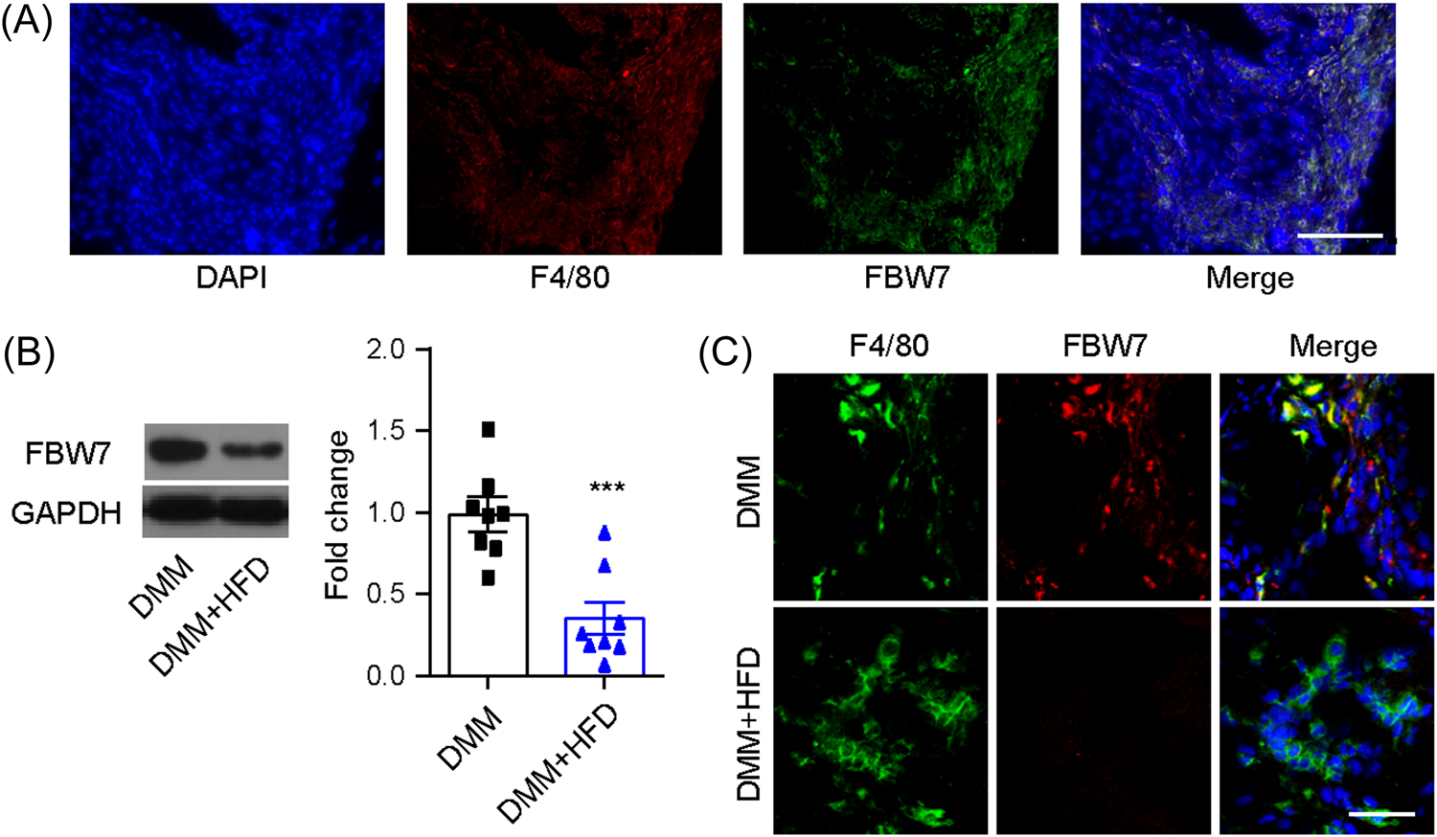
Involvement of macrophagic FBW7 in high‐fat diet (HFD)‐aggravated posttraumatic osteoarthritis outcomes. (A) Fluorescent immunohistochemistry examination of the cellular colocalization of FBW7 with macrophages Scale bar: 100 µm. (B,  C) Western blot and immunofluorescence results showed that FBW7 expression was significantly downregulated in destabilization of the medial meniscus (DMM) + HFD osteoarthritis (OA) tissues compared with the DMM group. Scale bar: 50 µm. *N* = 8, unpaired Student's *t* test, ***p < .001 compared with DMM.

To elucidate the potential mechanism underlying the aggravated posttraumatic OA outcomes, we speculated that a negative regulator of immunity (FBW7) may contribute to the regulatory process. We found that FBW7 specifically colocalizes with macrophages (Figure 3A). Furthermore, the expression level of FBW7 was significantly decreased in OA tissues from the DMM + HFD group compared with the DMM group, as confirmed by both western blot and immunofluorescence assays (Figure 3B,C), which further validated our earlier findings regarding colocalization.

### 3.4 Restoration of macrophagic FBW7 alleviates OA in vitro

**Figure 4:**
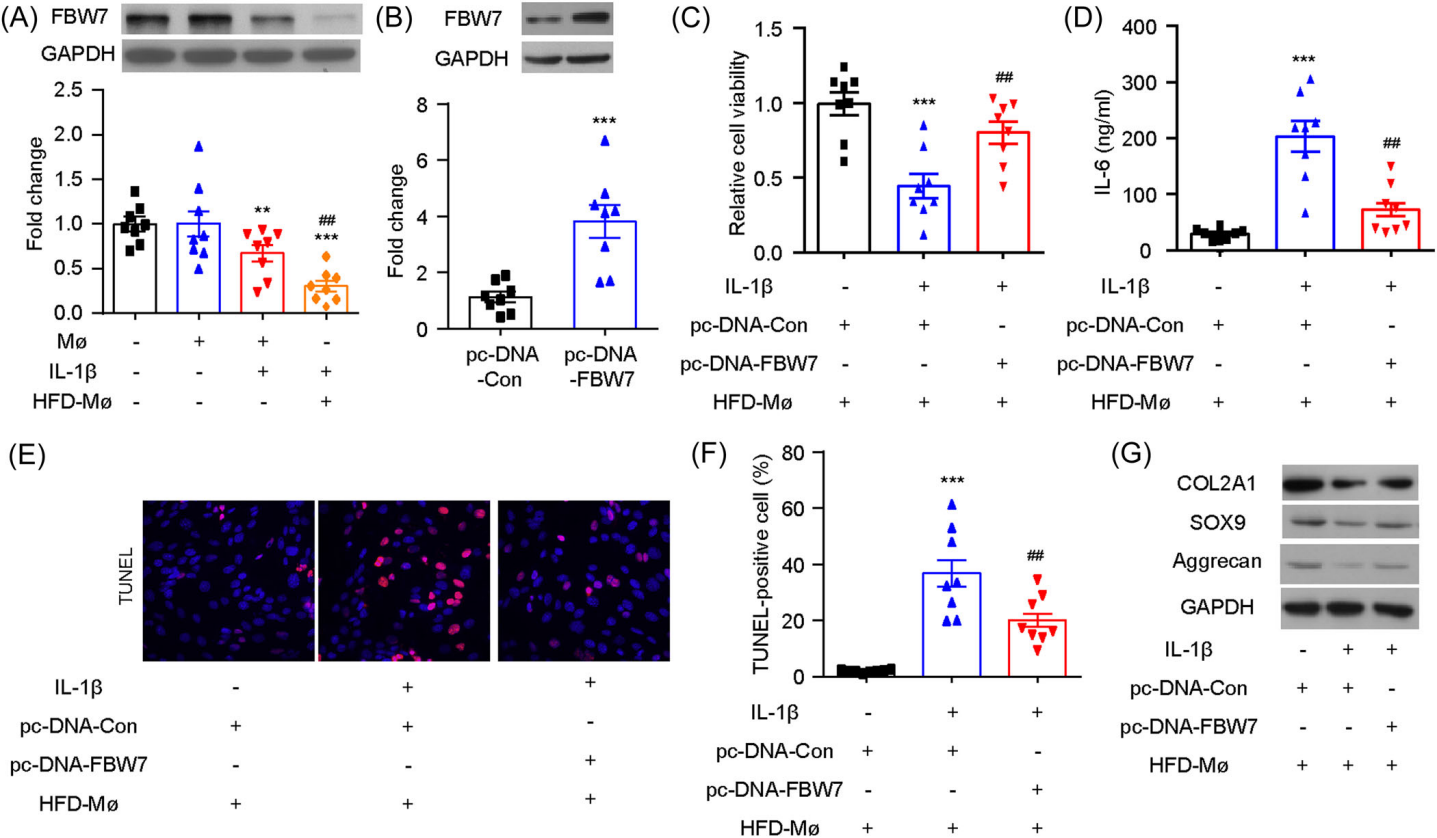
Macrophagic overexpression of FBW7 alleviates chondrocyte injury in vitro. Western blot results showed that FBW7 expression was significantly downregulated in macrophages isolated from high‐fat diet (HFD) mice upon interleukin‐1β (IL‐1β)‐induced chondrocyte injury. *N* = 8, one‐way analysis of variance (ANOVA) followed by Tukey post hoc test, **p < .01, ***p < .001 compared with Mø; ##*p* < .01 compared with Mø + IL‐1β. (B) The transfection efficiency was confirmed by Western blot analysis. ***p < .001 compared with pc‐DNA‐Con. *N* = 8, unpaired Student's *t* test, ***p < .001 compared with p‐DNA‐Con. (C) Cell viability analysis after FBW7 transfection (D) Expression analysis of IL‐6 after FBW7 transfection (E, F) TUNEL staining analysis after FBW7 transfection (G) Following transfection of FBW7, expression of ECM degradation markers was assessed by western blot analysis. Scale bar: 20 µm. *N* = 8, one‐way ANOVA followed by Tukey post hoc test, ***p < .001 compared with pc‐DNA‐Con + HFD‐Mø; ##*p* < .01 compared with IL‐1β + pc‐DNA‐Con + HFD‐Mø.

Consistent with the in vivo findings from the DMM model, our in vitro study using chondrocytes showed that stimulation with IL‐1β led to a further decrease in the expression of FBW7 in macrophages extracted from HFD mice (Figure 4A). These findings indicate that FBW7 may play a role in regulating macrophage immune responses and catabolism in chondrocytes. To further investigate the potential therapeutic effects of upregulating FBW7, we constructed a lentivirus carrying the FBW7 gene and confirmed its efficiency in transfection using western blot analysis (Figure 4B). The results of the CCK8 assay revealed that the upregulation of FBW7 could reverse the detrimental effects of IL‐1β on the viability of OA chondrocytes (Figure 4C). The IL‐6 enzyme‐linked immunoassay (ELISA) assay (Figure 4D) and TUNEL staining (Figure 4E,F) demonstrated that upregulation of FBW7 had beneficial effects on IL‐1β‐induced chondrocyte apoptosis. These findings were further confirmed with Western blot analysis (Figure 4G), which showed a decrease in extracellular matrix (ECM) degradation in the FBW7 overexpression group (Figure 4G). Overall, these findings indicate that the upregulation of macrophagic FBW7 is protective in OA by mitigating ECM degradation and decreasing inflammatory factor production in chondrocytes.

### 3.5 Involvement of FBW7 in OA development by upregulating the IL‐33/NF‐κB signaling pathway in macrophages

**Figure 5:**
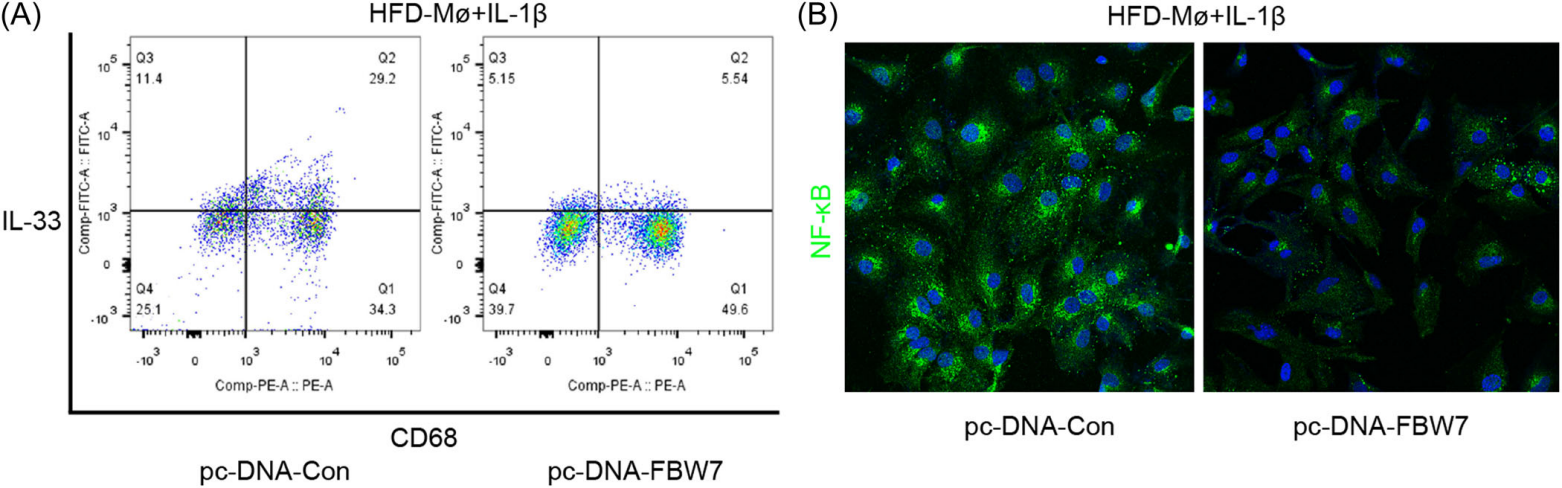
Macrophagic FBW7 modulates the IL‐33/NF‐κB signaling pathway. (A) Flow cytometry analysis of the percentage of IL‐33‐positive macrophagic cells after FBW7 transfection and interleukin‐1β (IL‐1β) stimulation. (B) Immunofluorescence staining images of macrophages showed the expression of NF‐KB. Scale bar: 100 µm.

FBW7 expression has been proposed to play a crucial role in regulating the nuclear factor‐kappa B (NF‐κB) signaling pathway. Macrophages are known to regulate inflammation through cytokine activity and phagocytosis. To investigate the impact of FBW7 expression on macrophages, we employed flow cytometry to measure the percentage of IL‐33+ macrophages. The results revealed that transfection with FBW7 led to the inactivation of macrophages and a reduction in their functional capabilities (Figure 5A). Moreover, immunofluorescence staining results indicated that the transfection of FBW7 potentially exerted inhibitory effects on the NF‐κB pathways (Figure 5B).

## 4 DISCUSSION

OA is the most prevalent form of arthritis, often leading to impaired physical function and a decreased quality of life.^20^, ^21^ Being overweight resulting from an HFD can increase mechanical pressure on the joints, leading to abnormal degradation of articular cartilage, bone loss, and chronic inflammatory reactions, which exacerbate OA symptoms.^22^ It has been replicated in various animal models, including rats,^10^, ^23^ rabbits,^24^ and guinea pigs.^25^ However, there is still a lack of comprehensive research on how HFD affects the aggravation of OA. In this study, we used both in vivo (DMM‐induced OA) and in vitro (IL‐1β‐induced OA) models to investigate the involvement of macrophages in the exacerbation of HFD‐related OA injuries. We found that decreased expression of FBW7 in macrophages contributes to the aggravation of HFD‐related arthritis injury.

Due to the limitations of clinical research, modern pharmacological experimental methods have increasingly turned to animal experiments to address scientific questions encountered in clinical settings. The animal models used in this study, involving HFD and OA after medial meniscectomy, are simple, reliable, and closely mimic clinical symptoms. They have been widely used in drug screening and pathogenesis research for related diseases. The HFD mimics the effects of Western diet structure on lipid metabolism, closely resembling real‐life situations. After 8 weeks of feeding HFD, the OARSI scores of the animals in the HFD group did not differ significantly from those in the normal diet group, and histopathological sections did not exhibit OA pathological characteristics. This indicated that HFD did not directly damage the knee joints, ruling out a direct impact. However, after DMM intervention, the pathological damage to the cartilage in the HFD group significantly aggravated. Therefore, we established that HFD negatively impacts the prognosis of arthritis. This model provides a solid experimental basis for our further exploration of the potential mechanisms underlying HFD as a risk factor for poor OA prognosis.

The coculturing of macrophages obtained from the aforementioned models with normal chondrocytes further verified the conclusions drawn from the in vivo experiments, confirming the critical role of macrophages in the aggravation process mentioned earlier. Macrophages play a crucial role in cartilage degradation and degeneration through their immune function.^26^ The inflammatory cytokines released by macrophages can cause matrix degradation and stimulate the proliferation and degeneration of synovial cells. They can also induce the production of inflammatory mediators by promoting the secretion of metalloproteinases in synovial cells and inhibiting or interfering with chondrocyte expression and phenotype.^27^ Our investigation of the potential participation of macrophages led us to focus on the FBW7 protein. FBW7 is an F‐box protein with seven tandem WD40 repeats.^28^ It has been extensively studied for its role in tumorigenesis and tumor development. Many molecules crucial in these processes are substrates of FBW7, including c‐myc, Notch1, c‐jun, and NF‐κB.^29^ FBW7 has also been implicated in the regulation of oxidative metabolism, glucose metabolism, and lipid metabolism in various disease models such as tumors, arteriosclerosis, and obesity.^30^ Our immunofluorescence colabeling studies revealed FBW7's colocalization with macrophages in joint tissues, suggesting the potential involvement of FBW7 in the aggravation of HFD‐induced arthritis.

Macrophages possess remarkable plasticity and heterogeneity, and their functions can adapt to different microenvironments. Our findings demonstrated a significant decrease in the expression of FBW7 in macrophages in the HFD group following DMM surgery, which was also confirmed in the in vitro model of OA. This indicates that FBW7 plays a role in regulating the functional homeostasis of resident macrophages in the joint cavity. Subsequent interventions targeting macrophage FBW7 further confirmed its role in the aggravation of HFD‐induced arthritis, thereby providing a new target for intervention and treatment of related diseases. Further mechanistic studies revealed that FBW7 regulated the expression of IL‐33 and the polarization of NF‐κB, along with other immune‐related phenotypes. This provides mechanistic evidence linking the abnormal expression of FBW7 in macrophages to the exacerbation of HFD‐induced OA prognosis.

HFD can contribute to the etiology and prognosis of arthritis through various factors. Here, we have uncovered the role of HFD in the poor prognosis of knee arthritis, particularly through its impact on macrophages. We have demonstrated that alterations in the macrophage FBW7 pathway, induced by HFD, serve as the underlying basis for the aggravation of pathological changes in knee OA. These findings deepen our understanding of OA and provide novel insights for a comprehensive consideration of disease phenotypes and the selection of more effective therapeutic targets. This contributes to the development of a comprehensive and efficient strategy for the management of knee OA.

### 4.1 Study limitation

The current study is limited by the absence of in vivo research. To address this limitation and further validate the cytological findings on FBW7l, we intend to perform in vivo experiments using appropriate models.

## AUTHOR CONTRIBUTIONS

**Lijun Duan**: Conceptualization; formal analysis; resources; writing—original draft; writing—review & editing. **Yuan Ma**: Conceptualization; formal analysis; methodology; writing—review & editing. **Chen‐Guang Feng**: Investigation; writing—review & editing. **Xing Yu**: Conceptualization; data curation; methodology; resources; writing—original draft; writing—review & editing.

## CONFLICT OF INTEREST STATEMENT

The authors declare no conflict of interest.

## ETHICS STATEMENT

Our experiment was supervised by the Animal Ethics Committee of Bayannaoer City Hospital (approved on 23 Feb 2022), Bayannaoer City, Inner Mongolia.

## Data Availability

The datasets used and/or analyzed during the current study are available from the corresponding author on reasonable request.
